# Micro-Drilling of Polymer Tubular Ultramicroelectrode Arrays for Electrochemical Sensors

**DOI:** 10.3390/s130506319

**Published:** 2013-05-14

**Authors:** Jan Kafka, Steen Skaarup, Oliver Geschke, Niels B. Larsen

**Affiliations:** 1 Department of Micro- and Nanotechnology, DTU Nanotech, Technical University of Denmark, Ørsteds Plads, Building 345Ø, DK-2800 Kongens Lyngby, Denmark; E-Mail: jk@inmoldbiosystems.com; 2 Department of Chemistry, Technical University of Denmark, Kemitorvet, Building 206, DK-2800 Kongens Lyngby, Denmark; E-Mail: skaarup@kemi.dtu.dk; 3 Aquaporin A/S, Ole Maaløes Vej 3, DK-2200 Copenhagen N, Denmark; E-Mail: oge@aquaporin.dk

**Keywords:** micro-drilling, PEDOT, poly(3,4-ethylenedioxythiophene), tubular electrode, ultramicroelectrode, TOPAS, microfluidic system, electrochemical detection, hydrogen peroxide, potassium ferro-/ferricyanide, finite element modeling

## Abstract

We present a reproducible fast prototyping procedure based on micro-drilling to produce homogeneous tubular ultramicroelectrode arrays made from poly(3,4-ethylenedioxythiophene) (PEDOT), a conductive polymer. Arrays of Ø 100 μm tubular electrodes each having a height of 0.37 ± 0.06 μm were reproducibly fabricated. The electrode dimensions were analyzed by SEM after deposition of silver dendrites to visualize the electroactive electrode area. The electrochemical applicability of the electrodes was demonstrated by voltammetric and amperometric detection of ferri-/ferrocyanide. Recorded signals were in agreement with results from finite element modelling of the system. The tubular PEDOT ultramicroelectrode arrays were modified by prussian blue to enable the detection of hydrogen peroxide. A linear sensor response was demonstrated for hydrogen peroxide concentrations from 0.1 mM to 1 mM.

## 1. Introduction

Microelectrodes are widely used as transducers in electrochemical sensors. The most common types are disc, cylinder, ring, and band shaped microelectrodes [1–3]. Tubular electrodes are a special type of band electrodes, as the length of electrodes is much larger than their width [4]. In addition to the advantages of common microelectrodes, such as fast establishment of a steady-state signal, enhanced mass transport at the electrode boundary, and an increased signal-to-noise ratio [5], tubular electrodes are well suited for integration into a microfluidic system as they do not disturb the flow of analytes since they are placed inside the channel sidewall. Even though tubular electrodes have been known for a long time [6], a reproducible method for fabricating polymer tubular electrodes has not been successfully realized. Few research groups have published suitable fabrication methods and often with unknown or inhomogeneous electrode dimensions on the sub-micrometer length scale. Deformation or degradation of the electrode material were observed during or after the fabrication, resulting in irregular electrode dimensions, independent of the fabrication method, e.g., drilling [7], laser cutting [8,9], or punching [8] and electrode material, e.g., gold [7], carbon paste [8], or diamond [9].

The higher conductivity and increased stability of conducting polymers have enabled their application as sensor electrodes over the last decades [10]. Polymer microelectrodes have been fabricated by a range of techniques, including photolithography [11], inkjet printing, [12,13] and hot embossing [14]. The lower conductivity and therefore higher electrical resistance of conducting polymers compared to noble metals restricts the areas of application as well as the useful electrode dimensions. Long band-shaped microelectrodes in particular suffer from low conductivity, which gives rise to a significant potential drop along the electrode. Tubular microelectrodes of equivalent dimensions can overcome the problems of varying potentials along the electrode since the conductive pathway to the electrode surface can be made essential two-dimensional (a conductive sheet) instead of one-dimensional (a conductive wire).

We have developed a fabrication method which allows a reproducible fabrication of polymer tubular electrode arrays. The electrodes were fabricated by micro-drilling through a layer of conductive poly(3,4-ethylenedioxythiophene) polymer (PEDOT), that had been spin-coated on both sides of a polymer carrier foil (cyclic olefin copolymer) and electrically insulated by a spin-coated layer of non-conductive polymer (polystyrene). Repeated drillings enabled the fabrication of Ø 100 μm tubular microelectrode arrays with an average electrode height of 0.37 ± 0.06 μm and with average processing times of 2 s per tubular electrode. The electrochemical response of different arrays towards potassium ferrocyanide showed high consistency. Functional microelectrode arrays were employed for the detection of hydrogen peroxide after a modification of the working electrodes with Prussian blue.

## 2. Experimental Section

### 2.1. Polymer Stack Fabrication

Polymer stacks for micro-drilling used a polymer foil (cyclic olefin copolymer, COC) as carrier substrate for the conductive (PEDOT) and non-conductive (polystyrene, PS) polymer thin film layers employed to form the tubular ultramicroelectrodes. The conductive polymer layers were firmly attached to the polymer foil substrate through an adhesion layer of spin-coated polystyrene, as detailed in the following: a layer of PS was applied onto an ethanol-cleaned Ø 6 cm circular piece of COC foil (TOPAS^®^5013, TOPAS Advanced Polymers, Frankfurt, Germany, thickness: 152 μm) by spin-coating 1 mL of 5 mg/mL PS in 1:1 (vol.) tetrahydrofuran/toluene at 1,000 rpm for 60 s using an acceleration of 500 rpm/s. Immediately after spin-coating of the PS, a layer of conductive polymer (poly(3,4-ethylenedioxythiophene) tosylate, PEDOT) was synthesized on top by spin-coating 0.9 mL of a freshly prepared solution of 2.150 mL CLEVIOS™ C-B50 (Heraeus Precious Metals, Leverkusen, Germany), 0.7 mL ultra-pure water (≥18 MΩ cm), 0.05 mL pyridine (99%, Sigma-Aldrich, Copenhagen, Denmark), and 0.08 mL CLEVIOS™ M V2 (>98%, Heraeus Precious Metals, Leverkusen, Germany) at 500 rpm for 90 s with an acceleration of 500 rpm/s. The substrate was afterwards baked at 65 °C for 15 min to increase the polymerization rate and to evaporate remaining solvents. Finally the sample was washed with deionized water and dried in a stream of nitrogen. The PEDOT layer thickness was about 0.3 μm, as determined by profilometry. The same procedure was repeated for the backside of the foil. After the final drying step an additional PS layer was applied on the backside PEDOT layer for electrical insulation: 1 mL of 100 mg/mL PS in 1:1 (vol.) tetrahydrofuran/toluene mixture was dispensed on one half of the foil (decentered). During spin-coating (750 rpm for 60 s with an acceleration of 250 rpm/s) PS got distributed unevenly on the sample, with a film thickness of the PS covered areas of about 26 μm as determined by profilometry. The covered half of the PEDOT layer was later used for electrode fabrication, while the uncovered PEDOT area provided electrical access.

For hydrogen peroxide detection, 50 mg/mL Prussian blue (PB, cat. 03899, Sigma-Aldrich, Copenhagen, Denmark) were added to the monomer mixture before spin-coating the backside PEDOT layer later used as working electrode. Prussian blue powder at 50 mg/mL in water was only partly soluble, so the suspension was ultrasonicated for 30 min followed by passive sedimentation for 12 h. The actual PB concentration in the supernatant of ≈12 mg/mL was determined by weighing after solvent evaporation. Only the supernatant phase was used for PEDOT/PB film synthesis.

### 2.2. Micro-Drilling and Device Assembly

To increase the mechanical stability before micro-drilling, the polymer stack was bonded to an injection molded COC through-hole chip system (TOPAS^®^5013) [15]. Bonding proceeded through the application of a patterned transfer adhesive (Intertronics, INTTA 106–100) to the chip system with cutouts matching the openings of the through-holes. The polymer stack was applied on the other side of the transfer adhesive, and the assembly gently pressed together. Through-holes were drilled into the polymer stack starting from the PS insulation layer into underlying chip through-holes using a Ø 100 μm drill (Kyocera Micro Tools cat. 226-0039.040, Kyocera Unimerco Tooling, Sunds, Denmark). The control software of the milling machine (Mini-Mill/3PRO, Minitech, GA, USA) was used to control the entire drilling process. Cutting speeds of 20 mm/min for the drilling process (downward movements) and 50 mm/min for upward and lateral movements were applied in order to fabricate an array of 10 electrodes in 20 s.

**Figure 1.**
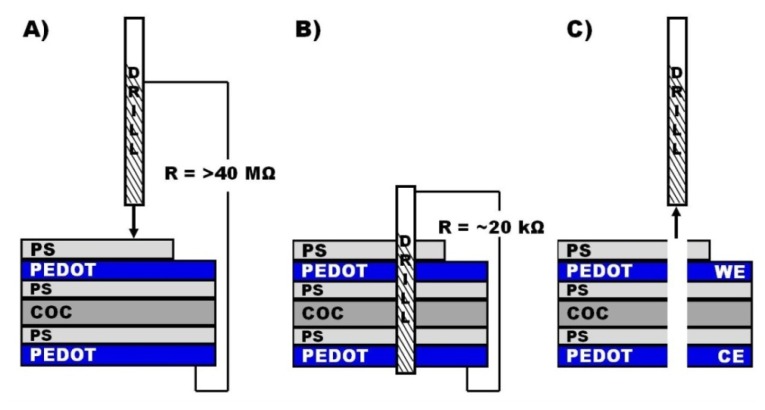
Tubular ultramicroelectrode fabrication in a polymer stack. (**A**) Both sides of an electrically insulating COC foil were spin-coated with a layer of polystyrene (PS, adhesion promoter) and conducting polymer (PEDOT). An additional PS layer was applied on one side to electrically insulate the underlying PEDOT layer. Micro-drilling through the stack showed a high resistance between the bottom PEDOT layer and the metal drill; (**B**) The monitored resistance dropped sharply when penetrating the bottom PEDOT layer, signaling completion of the drilling process; (**C**) Retraction of the drill left a cylindrical microchannel with integrated tubular working (WE) and counter (CE) electrodes.

Despite the mechanical support, the foils bent during drilling and the necessary drilling depth was larger than the sum of the polymer stack layer heights. By monitoring the electrical resistance between the drill and the lower PEDOT layer while drilling, the completion of the drilling through the polymer stack was determined by a drop in the electrical resistance from essentially disconnected to about 20 kΩ (Figure 1). Because of the conical end of the tool, drilling proceeded for additional 25 μm to achieve a homogeneous drilling shaft. During parameter optimization electrodes were fabricated at different feed rates on assemblies prepared with 60 s as well as 300 s PS spin times. Feed rate variations were achieved by changing the spindle speed (rotation rate of the tool), in order to keep the fabrication time for a 10 electrode array at 20 s. Feed rates from 0.5 μm/rev to 10 μm/rev were investigated. Higher feed rates caused distorted holes and were not further investigated. The samples were finally characterized by scanning electron microscopy (SEM) with respect to differences in the PEDOT deformation. Silver dendrites were deposited electrochemically at the tubular PEDOT electrodes to visualize the electro-active area by applying a potential of −0.3 V *vs.* Ag wire to the electrode in an aqueous solution of 0.1 mM AgNO_3_ for 10 s. The height of each electrode was determined at least at three different points along the PEDOT ring.

After drilling, a second through-hole chip was applied to seal the system. The aligned and gently attached systems were finally bonded in a press by applying 2.5 bar for 600 s at 50 °C. Electrical disconnection of the PEDOT layers at opposite sides of the foil was ensured by cutting off the rim of the foil.

### 2.3. Electrochemistry

Cyclic voltammetry on PEDOT electrodes was carried out in freshly prepared, nitrogen flushed 0.1 M potassium phosphate buffer (pH 7.0) containing 10 mM ferro-/ferricyanide of each species at scan rates from 5 mV/s to 500 mV/s in a potential range from −0.2 V to 0.6 V *vs.* Ag|AgCl|3M NaCl. For PEDOT/Prussian blue electrodes, 0.1 M potassium phosphate buffer (pH 7.0) was used. Amperometric detection of potassium ferrocyanide was realized on PEDOT electrodes at 0.5 V *vs.* Ag|AgCl|3M NaCl in 0.1 M potassium phosphate buffer (pH 7.0) at a flow rate of 100 μL/min (unless otherwise mentioned). After stabilization of a base current in phosphate buffer, potassium ferrocyanide in phosphate buffer was continuously injected for 300 s (analyzed concentration range: 1 mM to 300 mM), followed by a continuous injection of phosphate buffer. Amperometry on Prussian blue-modified PEDOT electrodes was carried out with hydrogen peroxide dissolved in phosphate buffer at 0 V *vs.* Ag|AgCl|3M NaCl.

### 2.4. Finite Element Modelling

The tubular electrodes were modelled in full 3D using the finite element modelling package COMSOL 4 (COMSOL AB, Stockholm, Sweden). The 370 nm high electrodes were modelled as perfect sinks of the analyte at an otherwise 200 μm thick mass transport insulated membrane. The membrane with Ø 100 μm through holes was placed as a constriction in the middle of a Ø 4 mm and approx. 2 mm long hollow cylinder. One end of the cylinder was defined as inlet with a constant flow rate and analyte concentration (diffusion constant of 8 × 10^−10^ m^2^/s [16]), the other end as an outlet with no viscous stress. The molar flux across the electrodes was converted to a current given that one electron is transferred for the reaction of each ferrocyanide ion.

## 3. Results and Discussion

### 3.1. Microelectrode Fabrication

Microelectrodes were fabricated by drilling through an electrically insulating polymer (COC) foil that was coated on both sides with a layer of polystyrene (PS), followed by a layer of PEDOT. The PS layers between the COC foil and the PEDOT layers were required to increase the adhesion between the COC foil and the PEDOT. An additional PS layer electrically insulated the upper PEDOT layer. Drilling through the whole assembly resulted in a cylindrical drilling shaft with a tubular PEDOT electrode integrated in the shaft sidewall and a large planar electrode placed at the end of the shaft (Figure 1). The drilling shaft acted as a microfluidic channel. Electrical access to the working electrode was realized by only partial application of an insulating PS layer on the upper PEDOT layer.

An adhesion layer of PS was required between the COC foil and each PEDOT layer to prevent delamination during the drilling process. Good adhesion of PEDOT to PS was demonstrated by unsuccessful attempts to remove spin-coated PEDOT from PS samples using strongly adhering office tape. Consequently, PS was used as intermediate layer between COC and PEDOT as well as for insulation of PEDOT on the working electrode. Due to the high fragility of thin PS samples, PS foil could not be used as replacement for the COC foil.

Alternative methods to improve the adhesion were explored, including partial integration of the COC and PEDOT by washing with a 1:1 (vol.) mixture of toluene and tetrahydrofuran as described earlier [17]. However, the observed increase in adhesion was insufficient to prevent delamination during the drilling process. Repeating the washing step to further promote adhesion caused substantial bending and damage to the foil.

### 3.2. Microelectrode Characterization and Optimization

**Figure 2.**
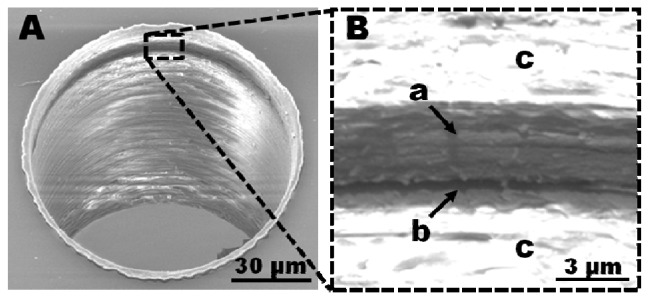
(**A**) SEM micrograph of a hole drilled through a polymer stack of PS/PEDOT/PS/COC foil, showing the integrated tubular PEDOT electrode as a black ring at the upper end of the drilling shaft; (**B**) Zooming in on the region of the black ring shows (a) deformed PEDOT around (b) the original PEDOT layer embedded in (c) the insulating PS polymer layers. Both micrographs are presented in a 30° tilted perspective.

The dimensions of the fabricated electrodes were visualized by scanning electron microscopy (SEM). Micrographs of the fabricated holes show tubular electrodes as a dark ring at the upper end of the drilling shaft (Figure 2(A)). A magnification of the region around the electrode showed distinctive areas inside the ring: a grey zone (a) around a deep black ring (b), surrounded by brighter zones (c) (Figure 2(B)). The conductive polymer is expected to show less charging during SEM imaging than the surrounding insulating polymer material, thus making it plausible that the black ring (Figure 2(B), area (b)) corresponds to the PEDOT layer. The grey zone around the black ring could originate in an accumulation of less conductive material, e.g., PEDOT smeared along the sidewalls during the drilling process (Figure 2(B)-a). The bright areas above and below the dark zone are most likely the pure non-conductive polymers COC and PS that charge strongly during SEM imaging.

Deformation of the electrode material has been observed by other research groups. Corti *et al.* described deformation after drilling into a thin gold layer deposited on a cylindrical insulating material (Lucite) and covered with epoxy-resin [7]. The gold layer was deformed along the drilling shaft sidewall and increased the effective electrode area. Konash *et al.* observed a smearing of carbon paste along the surrounding material upon physical contact with a punching tool as well as after laser ablation [8]. The observed similarities in our experimental results suggest that the PEDOT layer becomes deformed during the drilling process.

**Figure 3.**
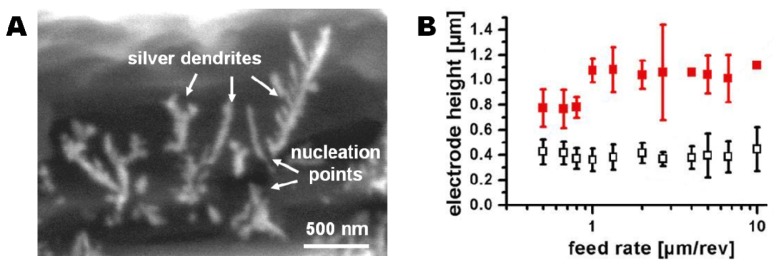
(**A**) SEM micrograph of electrochemically deposited silver dendrites on a tubular PEDOT electrode fabricated at a feed rate of 2.7 μm/rev. The electro-active height of the tubular electrode is determined from the vertical extent of the silver dendrites' nucleation points; (**B**) Measured electro-active PEDOT electrode heights as function of the feed rates for electrodes fabricated in polymer stacks produced using a solvated (open symbols) or dried (solid symbols) PS layer.

Smearing of the electrode area is not necessarily a problem if the smeared regions are electrically disconnected. Electrochemical deposition of silver dendrites enabled visualization of the electroactive electrode area (Figure 3(A)). Silver dendrites were only observed within a narrow band of the black ring that was initially considered to be highly conductive PEDOT material (Figure 2(B)-b). Even if the grey zone around the black ring (Figure 2(B)-a) consists of deformed PEDOT, these areas were not found to be electrochemically active. Therefore they will not participate in electrode processes. Analysis of the distance between the silver dendrite nucleation points across the active PEDOT area allowed determination of the actual electrode dimensions (Figure 3(B)). The solid markers show the measured electrode thickness resulting from drilling through a polymer stack where the PS adhesion layer was allowed to dry before spin coating and polymerization of the PEDOT layer. A minimum electrode thickness of 780 ± 84 nm was found for a set of electrodes fabricated with a feed rate of 0.8 μm/rev (Figure 3(B)). From previous experiments (data not shown) the PEDOT layer thickness for the applied set of spin-coating parameters was expected to be around 300 nm. Therefore the determined electrode heights indicate a significant PEDOT deformation during the drilling process. A range of processing parameters was investigated to identify the critical factors in minimizing smear. The analysis showed a major influence of two parameters as described in detail in the following sections: (1) The solvation state of the PS layer before application of the PEDOT polymerization solution, and (2) the feed rate during the drilling.

As discussed earlier, the application of a PS layer as adhesion promotor between the COC foil and the PEDOT layer was required to avoid delamination. The initially fabricated set of electrode arrays used PS layers spun for 300 s to ensure evaporation of the majority of the solvents, and thereby a distinct separation of the PS layer from the subsequently applied PEDOT layer. The time was determined by a weight controlled dry-spinning process: A Ø 6 cm TOPAS foil was spin-coated with a layer of PS and spin dried for another 300 s. The weight of the coated foil was measured after the first 60 s of spin-coating and subsequently after every 30 s of the spin drying process. Due to evaporation of solvent a non-linear mass decrease of about 4 mg in total was observed over the whole period of 300 s. A subsequent heating of the foil to 75 °C for 2 min to evaporate any remaining solvent reduced the weight only by another milligram, indicating a nearly finished evaporation process after 300 s at room temperature. As shown in Figure 3(B) (solid markers), drilling through polymer stacks with pre-dried PS layers resulted in electrodes with a minimum electroactive height of 780 ± 84 nm at a feed rate of 0.8 μm/rev.

The use of a partly solvated PS layer during application of the PEDOT layer might lead to layer intermixing. Strong intermixing would lead to undesirable broadening of the PEDOT layer thickness. In contrast, small degrees of intermixing could increase the mechanical strength of the PEDOT/PS interface and thus favorably reduce smear during drilling. The dominant effect was tested by producing a second set of electrodes using equivalent drilling conditions (feed rate of 0.8 μm/rev) on polymer stacks prepared with a solvated PS layer (PS spin coating time of 60 s). The resulting electrode height was 0.37 ± 0.12 μm (Figure 3(B), open symbols), *i.e.*, less than half the height of the electrodes produced using a dried PS layer and close to the expected height of the PEDOT layer (≈300 nm). Thus, the dominant effect is the improvement in mechanical strength after PS/PEDOT intermixing.

Variation of the feed rates applied during drilling had no significant influence on the effective electrode height. Electrodes were produced with feed rates from 0.5 μm/rev to 10 μm/rev on the two different types of polymer stacks (dried or solvated PS layer before PEDOT layer application). Feed rates above 10 μm/rev caused distorted drilling shaft profiles and were not further investigated. All fabricated electrodes could be largely divided into two sets of electrode dimensions: Electrodes fabricated on polymer stacks prepared using a solvated PS film (PS spin time: 60 s) had electroactive heights of 0.40 ± 0.11 μm while electrodes fabricated on polymers stacks using dried PS layers (PS spin time: 300 s) had electroactive heights of 0.97 ± 0.22 μm (Figure 3(B)).

For sensor applications, a high reproducibility of the electrode fabrication is important. Since the average electrode height was independent of the applied feed rate, the homogeneity of the electrodes was taken as indicator for optimal drilling parameters. The lowest electrode height deviation of 0.37 ± 0.06 μm was observed for electrodes drilled with a feed rate of 2.7 μm/rev on polymer stacks produced with solvated PS layers, so these fabrication parameters were used for all electrochemical systems.

### 3.3. Electrochemical Setup and Electrode Characterization

**Figure 4.**
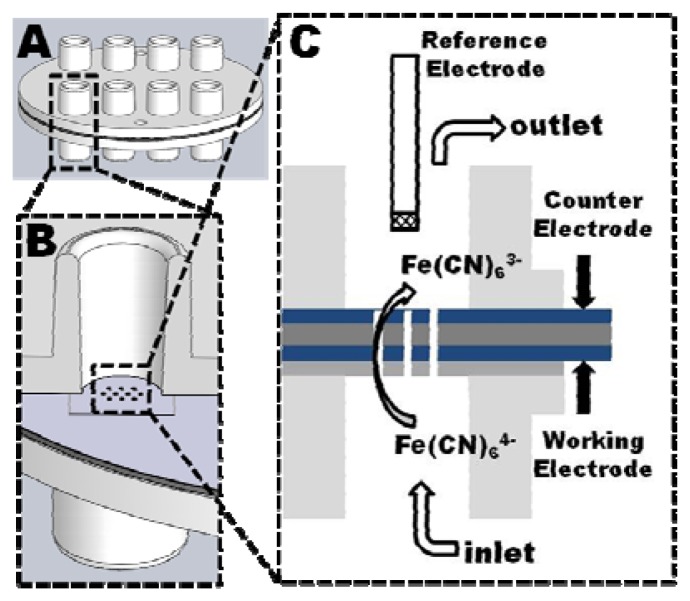
Electrochemical setup of the assembled device. (**A**) Schematic of the analysis device consisting of a polymer stack bonded between two polymer chips with through-holes for connection to pumps and potentiostats; (**B**) Cross section through a single analysis unit having 10 tubular microelectrodes in the polymer stack; (**C**) Electrochemical analysis uses a Ag|AgCl|3M NaCl reference electrode inserted into the upper through-hole, and tubing (not shown) connected to the lower and the upper through-hole as inlet and outlet, respectively. The tubular working electrodes and the planar counter electrode are electrically contacted by spring-loaded pins through neighboring through-holes.

Before drilling of the tubular electrodes, the polymer stacks were bonded to an injection molded COC multi through-hole chip to increase the mechanical stability of the stack. After drilling a second through-hole chip was bonded to the other side of the polymer stack to seal the system by applying a silicone based adhesive tape (Figure 4(A)).

The through-holes of the chips were designed as female Luer-connector (Figure 4(B)), allowing an easy connection of the assembled analysis device via male-Luer connectors to a syringe pump. A Ag|AgCl|3M NaCl reference electrode was inserted in the opposite through-hole which was simultaneously used as outlet. The working electrodes (tubular electrode arrays) and counter electrode (planar PEDOT layer at the outlet) were both electrical connected through spring loaded metal pins which were inserted into the neighboring through-holes (Figure 4(C)).

**Figure 5.**
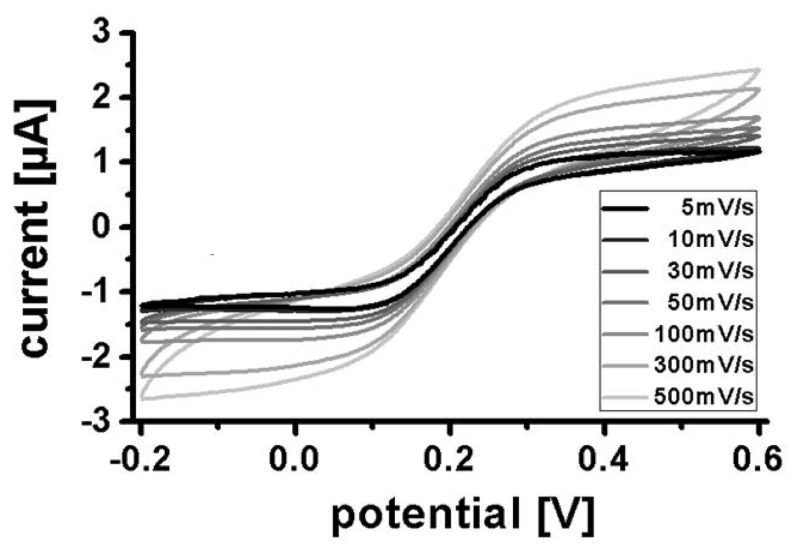
Cyclic voltammograms of 10 mM potassium ferrocyanide/10 mM potassium ferricyanide in 0.1 M potassium phosphate buffer using an array of 10 tubular PEDOT ultramicroelectrodes of diameter 100 μm, a PEDOT counter electrode, and a Ag|AgCl|3M NaCl reference electrode.

Cyclic voltammograms were recorded in potassium phosphate buffer containing 10 mM ferro-/ferricyanide at scan rates from 5 mV/s to 500 mV/s (Figure 5). At lower scan rates (<100 mV/s) the voltammograms have a typical ultramicroelectrode sigmoidal shape with well-defined mass transport limited current plateaus for oxidation and reduction. At higher scan rates (≥100 mV/s) the oxidation and reduction plateaus become less well defined. This could be related to ohmic resistance in the very thin conductive polymer leads to the electrode. However, similar variations in shape with scan rates have also been observed on ultramicroelectrodes made from gold or platinum of much higher conductance [18].

### 3.4. Amperometric Detection of Potassium Ferrocyanide

**Figure 6.**
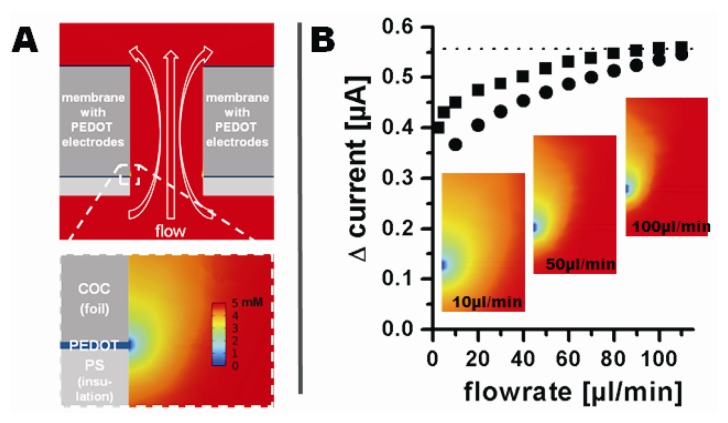
(**A**) Finite element modelling of the redox active species concentration at steady state. The upper figure shows a cross-section of the 3D model of a single Ø 100 μm tubular PEDOT electrode. The COC foil (dark gray bars) carries the PEDOT electrodes (blue) and the PS insulating layers (light gray) surrounded by an aqueous solution of 5 mM redox active species (red color). The liquid flow direction is indicated by the hollow white arrows. The lower figure is a zoom on the tubular electrode region, where redox processes leads to total depletion of the species at the electrode surface, resulting in gradual compound depletion at increasing distance to the electrode; (**B**) Modelled (circles) and measured (squares) current dependency on analyte flow rate. Inserts: Finite element modelling results for the analyte concentration near the electrode surface for flow rates of 10 μL/min, 50 μL/min, and 100 μL/min at steady state (same color legend as in A).

The influence of the flow rate on the measured current was analyzed before recording the amperometric response of the electrodes towards different ferrocyanide concentrations. Oxidation currents in the presence or absence of 5 mM ferrocyanide in 0.1 M phosphate buffer were recorded at flow rates from 2.5 μL/min to 110 μL/min at 500 mV *vs.* Ag|AgCl|3M NaCl. Flow rates below 2.5 μL/min could not be investigated due to increasing disturbance caused by the syringe pump. The current change was defined as the difference between the signal in absence and in presence of ferrocyanide at each flow rate. An increase of the flow rate from 2.5 μL/min to 100 μL/min resulted in a non-linear increase in the oxidation current from 400 nA to 557 nA. A further increase in flow rate did not significantly increase the current (Figure 6(B)). The decreasing current with decreasing flow rate indicates a mass transfer limited regime.

The electrode behaviour was modelled using the finite element modelling package COMSOL 4. Figure 6(A) shows a cross-section of the model through the channel and a magnification of the surrounding of an electrode. The COC foil is shown as dark gray, the electrodes as blue, and the insulating PS layer as light gray. The analyte flow is indicated by the white hollow arrows. The different colors of the analyte around the electrode represent different analyte concentrations and therefore the modelled depletion layer at steady state. Figure 6(B) compares the current predicted by modelling (converted from the calculated analyte flux through the electrode surface) to the experimentally obtained current changes at corresponding flow rates. The modelled and experimentally obtained currents are found to be in very good agreement, especially at higher flow rates.

**Figure 7.**
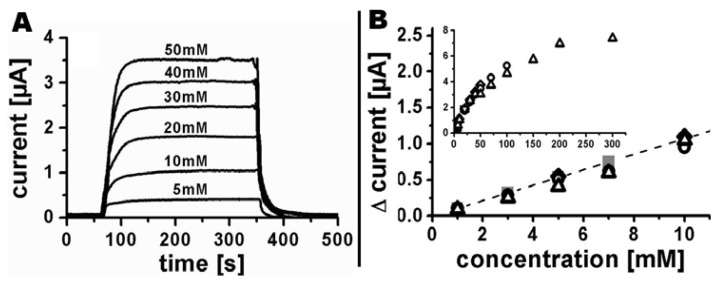
(**A**) Amperometric response of a ten Ø 100 μm tubular PEDOT electrode array to different potassium ferrocyanide concentrations in 0.1 M potassium phosphate buffer, at a flow rate of 100 μL/min and an electrode polarization of 500 mV *vs.* Ag|AgCl|3M NaCl; (**B**) Current change versus potassium ferrocyanide concentration for the linear response regime in the range from 1 to 10 mM. Insert: Current change for all analyzed potassium ferrocyanide concentrations. Different black, open symbols represent measurements with independent systems, closed gray squares connected with the dashed line are the results predicted by finite element modelling.

Amperometric detection of different ferrocyanide concentrations was realized using a flow rate of 100 μL/min. Initially, the base current in phosphate buffer was recorded until signal stability was achieved, followed by injections of phosphate buffer containing ferrocyanide for 300 s. Afterwards pure phosphate buffer was injected again to ensure the reestablishment of the initial base current (Figure 7(A)). A linear current increase was found for concentrations up to 10 mM (Figure 7(B)). A further increase in concentration up to 300 mM showed a nonlinear current response (insert Figure 7(B)), most likely caused by increasing electrostatic interactions and ion-complexations. The current responses of four independent systems (different symbols in Figure 7(B)) showed very little variation, which demonstrates the high reproducibility of the fabrication process. Each sensor was used for 3.5 h on average for detection of different ferrocyanide concentrations without any indications of loss in sensitivity. Injections of phosphate buffer containing 5 mM ferrocyanide resulted in comparable sensor responses at the beginning, during, and at the end of each measurement series. Longer periods were not investigated during this study. However, the long-term stability of PEDOT in air or in aqueous solution within a wide range of pH values was previously studied in detail by Winther-Jensen and West [19] who observed small conductivity changes for storage in air for many months and almost constant conductance in aqueous environments in the range from pH 5–10 for shorter time periods.

Results from the finite element model for the linear response regime from 1 to 10 mM are presented in Figure 7(B) as gray solid squares connected by a dashed line. The slope of the modelled current-concentration dependency is 0.107 μA/mM which is very close to the experimentally obtained standard curve with a slope of 0.102 μA/mM.

### 3.5. Amperometric Detection of Hydrogen peroxide

Prussian blue (PB) is known to be an excellent and specific mediator for hydrogen peroxide reduction [20]. Hydrogen peroxide sensitive electrodes were fabricated by adding PB to the polymerization solution prior to spin-coating the PEDOT later used as working electrode. This led to embedding of PB into PEDOT during polymerization of the latter. The PEDOT layer used as counter electrode was made without any additives and therefore did not contain any PB.

**Figure 8.**
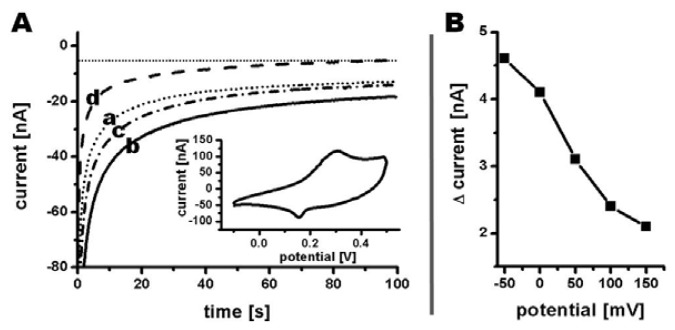
(**A**) Amperometric responses of a ten Ø 100 μm tubular PEDOT/PB electrode array. Traces (a) through (c) were measured sequentially using (a) 0.1 M potassium phosphate, (b) 0.5 mM hydrogen peroxide in 0.1 M potassium phosphate, and (c) 0.1 M potassium phosphate at 0 mV *vs.* Ag|AgCl|3M NaCl. Trace (d) was calculated as the difference between (a) and (b). Insert: Cyclic voltammogram recorded in 0.1 M potassium phosphate at a scan rate of 10 mV/s using a ten Ø 100 μm PEDOT/PB electrode array, a PEDOT counter electrode, and a Ag|AgCl|3M NaCl reference electrode; (**B**) Voltammogram within the range from −0.05 V to 0.15 V *vs.* Ag|AgCl|3M NaCl in the presence of 0.5 mM hydrogen peroxide.

A cyclic voltammogram at 10 mV/s was recorded in phosphate buffer showing an oxidation peak at 310 mV as well as a reduction peak at 150 mV *vs.* Ag|AgCl|3M NaCl (Figure 8(A) insert). Since the reduction current for hydrogen peroxide increases using an overpotential, the signal strength at lower potentials was investigated [21]. A voltammogram from 150 mV to −50 mV was recorded by measuring the amperometric currents at corresponding potentials in phosphate buffer before and after recording the signal in phosphate buffer containing 0.5 mM hydrogen peroxide (Figure 8(A)). The current difference between the initial measurement and the measurement in presence of hydrogen peroxide were used for creating a voltammogram (Figure 8(B)), with all currents measured at a time of 100 s after liquid exchange. An increase in reduction current was determined from 2.1 nA at 150 mV to 4.1 nA at 0 mV and even further to 4.6 nA at −50 mV (Figure 8(B)). Even though the measured current increased for potentials more negative than 0 mV, a reduction potential of 0 mV was chosen for the following amperometric detection of different hydrogen peroxide concentrations due to almost complete elimination of interferences when measuring on biological samples at this potential [22].

**Figure 9.**
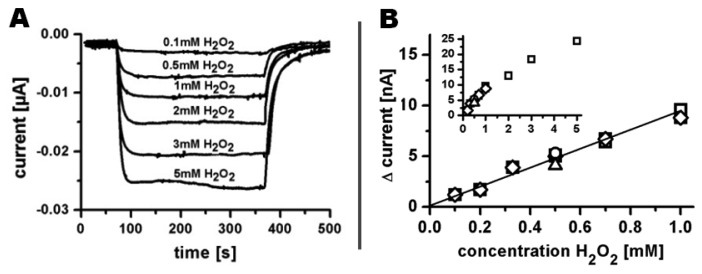
(**A**) Amperometric response of a ten Ø 100 μm PEDOT/PB electrode array towards different hydrogen peroxide concentrations diluted in 0.1 M potassium phosphate buffer at a flow rate of 100 μL/min and an electrode polarization of 0 mV *vs.* Ag|AgCl|3M NaCl; (**B**) Linear response regime of independent PEDOT/PB electrode arrays (different open symbols) towards hydrogen peroxide within the range from 0.1 mM to 1 mM. Insert: Current response of the electrode arrays versus hydrogen peroxide concentration for a range up to 5 mM hydrogen peroxide in 0.1 M potassium phosphate.

Amperometric detection of hydrogen peroxide was realized by recording a base current in phosphate buffer at 100 μL/min until steady state, followed by continuous injection of phosphate buffer containing hydrogen peroxide for 300 s (Figure 9(A)). Afterwards the system was flushed with phosphate buffer again to ensure initial electrode conditions before injecting buffer containing a different hydrogen peroxide concentration. Injections of hydrogen peroxide concentrations from 0.1 mM to 5 mM resulted in an increasing reduction current from 1 nA to 24 nA, respectively. Higher concentrations were not analyzed due to beginning signal instability already visible at 5 mM. A linear response regime of the sensor was observed for a concentration range from 0.1 mM to 1 mM with a sensitivity of 8.6 nA/mM.

## 4. Conclusions/Outlook

Successful fabrication of homogeneous PEDOT tubular ultramicroelectrode arrays and their electrochemical functionality was demonstrated. The right choice and arrangement of the supporting non-conductive polymers for a thin layer of PEDOT as well as optimization of the fabrication parameters allowed the use of micro-drilling as a fast prototyping strategy to produce Ø 100 μm tubular electrodes with an average height of 0.37 ± 0.06 μm. A comparison of experimental electrochemical results to results obtained from a finite element model showed good agreement, and supported the excellent functionality of the tubular polymer electrode arrays for voltammetric applications (at least at lower scan rates) as well as amperometric applications. However, the low conductivity of PEDOT compared to metal does not allow the application of the electrode arrays for analytical methods where fast potential changes are involved (e.g., cyclic voltammetry at very high scan rates). This excludes selected applications of the fabricated conductive polymer electrodes but still leaves a wide field of operational possibilities within the field of sensors. Simple electrode modifications allow the fabrication of sensor electrodes, e.g., for amperometric detection of an analyte where no potential changes are involved. This concept was demonstrated by hydrogen peroxide detection after a prussian blue modification of the PEDOT electrodes, with a linear current response for hydrogen peroxide concentrations from 0.1 mM to 1 mM.
